# A Preliminary Investigation of the Relationship between Motivation for Physical Activity and Emotional and Behavioural Difficulties in Children Aged 8–12 Years: The Role of Autonomous Motivation

**DOI:** 10.3390/ijerph17155584

**Published:** 2020-08-03

**Authors:** Erin Farmer, Nicole Papadopoulos, Chloe Emonson, Ian Fuelscher, Caterina Pesce, Jane McGillivray, Christian Hyde, Lisa Olive, Nicole Rinehart

**Affiliations:** 1 Deakin Child Study Centre, School of Psychology, Faculty of Health, Deakin University, Geelong, VIC 3220, Australia; erin.farmer@deakin.edu.au (E.F.); chloe.emonson@deakin.edu.au (C.E.); jane.mcgillivray@deakin.edu.au (J.M.); c.hyde@deakin.edu.au (C.H.); nicole.rinehart@deakin.edu.au (N.R.); 2 Centre for Social and Early Emotional Development (SEED), School of Psychology, Faculty of Health, Deakin University, Geelong, VIC 3220, Australia; ian.fuelscher@deakin.edu.au (I.F.); lisa.olive@deakin.edu.au (L.O.); 3 Department of Movement, Human and Health Sciences, University of Rome “Foro Italico”, 00135 Rome, Italy; caterina.pesce@uniroma4.it; 4 The Institute for Mental and Physical Health and Clinical Translation (IMPACT), School of Medicine, Deakin University, Geelong, VIC 3220, Australia

**Keywords:** physical activity, children, motivation, autonomous motivation, mental health

## Abstract

While motivation for physical activity (PA) and PA participation have been linked, research on the relationship between motivation for PA and mental health outcomes is scant, with studies involving children largely underrepresented. Grounded in self-determination theory, this cross-sectional study aimed to determine whether autonomous motivation versus external motivation (a form of controlled motivation) for PA is associated with fewer emotional and behavioural difficulties and higher levels of PA in children. A sample of 87 children (aged 8–12 years) were recruited from five primary schools in Victoria, Australia. An adapted version of the Behavioural Regulation in Exercise Questionnaire (BREQ) was used to measure motivation for PA and structured parent-report questions were used to assess moderate-to-vigorous PA (MVPA) levels. Parents also completed the Strengths and Difficulties Questionnaire (SDQ) to measure children’s emotional and behavioural difficulties. Children’s autonomous motivation was associated with fewer emotional and behavioural difficulties (*β* = −0.25, *p* = 0.038) and higher levels of MVPA (*β* = 0.24, *p* = 0.014). These results indicate autonomous motivation is associated with improved mental health outcomes and higher levels of PA in children. Thus, PA interventions that promote autonomous motivation may enhance children’s mental health compared to interventions that promote mainly controlled forms of motivation.

## 1. Introduction

It is estimated that 14% of Australian children and adolescents have experienced a mental health disorder such as Attention-Deficit/Hyperactivity Disorder (AD/HD; characterised by inattentive, hyperactive and impulsive behaviours [1]), anxiety (characterised by excessive worry or concern [2]) or depression (characterised by sadness, irritability, feelings of worthlessness, hopelessness [3]) in the last 12 months [4]. The consequences of mental health difficulties can be profound, impacting quality of life, education, employment, relationships and increasing the risk of suicide [5]. Identifying and enhancing childhood protective factors is one of the best methods of preventing and/or reducing the life-long impact of mental health disorders [6]. Physical activity (PA) is one such protective factor [7], where PA is defined as “any type of muscular activity that increases energy expenditure substantially” [8] (p. 197). Meta-analytic research and systematic reviews have found positive effects of PA for a number of different mental health disorders such as preventing and reducing symptoms of depression and anxiety [9,10,11]. PA also has benefits for positive mental health, including self-esteem [9], life satisfaction and happiness [12], and health-related quality of life (HRQoL) [13].

Effective public health promotion of PA is therefore paramount, not only for physical health but also mental health. As such, research into factors that explain PA behaviours and associated mental health outcomes has increased in recent years. Studies exploring motivation for PA, based on self-determination theory [14], conceptualise motivation as either autonomous (more self-determined) or controlled (less self-determined). Autonomous motivation includes actions made for inherent satisfaction or enjoyment (intrinsic motivation), actions that align with aspects of the self (integrated regulation) or actions that are valued (identified regulation). In contrast, controlled motivation induces actions to enhance ego, or to suppress feelings of guilt, shame, and anxiety (introjection), or to meet external demands or rewards (external regulation) [15]. 

Both motivation types direct behaviour, but according to self-determination theory, autonomous motivation results in stronger and longer lasting behaviour change than controlled motivation [16,17]. As such, autonomous motivation has been shown to be associated with higher levels of PA in children and adolescents [18,19,20,21,22,23]. Furthermore, motivation towards PA is emerging as an important factor for mental health. According to self-determination theory, when an individual’s motivation is autonomous they experience better behavioural outcomes as well as improved wellbeing and mental health [17,24,25]. For example, increases in autonomous motivation, relative to controlled motivation, have been found to predict fewer depressive symptoms in adults [26] and higher levels of positive affect in adolescents [27]. This positive effect on mental health is explained by a subtheory of self-determination theory, basic needs theory [17], which posits that people who feel a sense of mastery (competence), in control (autonomy) and connected to others (relatedness) will be more likely to have autonomous motives and improved mental health outcomes. 

Controlled motivation has been found as a weaker predictor of PA relative to autonomous motivation and may also contribute to an increased risk of mental health problems via the thwarting of the basic needs [24,26]. Ng et al.’s [28] meta-analysis of 166 studies found controlled forms of motivation to demonstrate a significant positive association with depression and anxiety. Further, higher levels of controlled motivation have been associated with lower levels of positive affect and lower subjective wellbeing in adults and adolescents [27,29]. While the balance of evidence suggests that controlled motivation may be detrimental to fostering positive mental health, not all studies have reported a negative relationship between controlled motivation and mental health [30]. 

Notwithstanding a growing evidence base, studies involving child samples remain largely underrepresented, despite the common finding that self-determined motivation for PA decreases with age [18]. The age at which the change begins is yet to be agreed upon empirically but is believed to take place from late childhood to mid-adolescence [18]. Some studies report the decline to begin around the age of 12 years [31]. In contrast, a three-year longitudinal study of students aged 10–13 years (*n* = 134), reported no overall changes in intrinsic (autonomous) motivation [21], suggesting that changes may not appear until after age 13. Despite this general belief that motivation for PA adjusts significantly between childhood and adolescence, the lack of studies including children under the age of 10 years makes it difficult to know when and how to intervene for children. 

Emerging evidence suggests that motivation for PA independently contributes to improved mental health outcomes [25]. Leveraging protective factors in childhood can prevent, or at least reduce, the potential life-long impact of mental health problems. Despite this, the small number of studies into motivation for PA that have attended to mental health have been mostly conducted with adults or adolescents, not children [32]. Given the developmental changes that take place across childhood, adolescence and into adulthood [33], adolescent research is not generalisable to children. Furthermore, evidence indicates that motivation for PA may fluctuate between childhood and adolescence [34], reinforcing the need for more research in children.

Consistent with the hypotheses drawn from self-determination theory that autonomous motivation will predict mental health outcomes and participation in PA, this study had two aims. The primary aim was to explore the degree to which autonomous versus controlled motivation is associated with fewer emotional and behavioural difficulties, both well-established precursors of mental health problems, adjusting for age, gender and PA levels. The secondary aim was to explore the degree to which autonomous versus controlled motivation may be associated with parent-reported PA levels in a sample of Australian children, adjusting for age and gender. 

## 2. Materials and Methods 

### 2.1. Participants

Five schools in Victoria, Australia participated in the study, from which 87 children (43 boys, 44 girls, *M*_age_ = 10.02 years, age range: 8–12 years) and their parents were recruited from 35 classes. Cross-sectional data was collected in the baseline survey of the Australian Joy of Moving (AJoM) study, a pilot study evaluating the feasibility and impact of a psychoeducational classroom-based PA program to foster both movement and mental health in children. For inclusion in the present study, participants needed to be enrolled in a mainstream primary school in Victoria where both the principal and classroom teacher had consented to participating in the AJoM study. In line with the feasibility objectives of the pilot, each participating school was able to select a whole-school-participation approach or restrict participation to select classes. At the discretion of the school principal and classroom teachers, two schools selected a whole-school approach and three schools ran the program in specific classes (School 1: grades Prep, 3–6; School 2: grades Prep-5; School 3: grades 5 and 6). All children in participating classes were invited to participate in the AJoM pilot study. However, data was only collected from children enrolled in grades 3–6. No other exclusion criteria were applied (see Table 1 for participant characteristics and descriptives). Families of children attending pilot schools were recruited and asked to complete a baseline survey four to six weeks before the commencement of the AJoM intervention. As part of the baseline survey, basic child demographic information (age, gender, school grade and any health conditions previously diagnosed by a medical professional) was collected. This study was approved by the Human Research Ethics Committee of Deakin University, Melbourne, Australia (2017-347) and the Victorian Government, Department of Education and Training. Informed consent was obtained from parents in accordance with the Declaration of Helsinki. 

### 2.2. Measures

#### 2.2.1. Physical Activity

Moderate-to-vigorous PA (MVPA) levels were assessed via structured parent-report questions developed specifically for the present study. Subjective reporting of lighter intensity PA has shown poor reliability in child samples [8] and was therefore excluded from measurement. Parents estimated the total amount of time their children usually spend engaging in vigorous PA in a typical week in hours and minutes, where vigorous PA was defined as “activities such as jogging and cycling that cause your child to become quite breathless”. Parents were then asked to make the same estimations for moderate PA defined as “activities such as walking the dog or play that cause your child to become a little bit breathless”. Both PA estimates were added to obtain the total number of minutes spent engaging in MVPA in a typical week. 

#### 2.2.2. Motivation

Motivation toward PA was assessed using an adapted version of the Behavioural Regulation in Exercise Questionnaire (BREQ) [35] modified specifically for children [36]. The adapted BREQ [36] consists of 12-items with four subscales assessing external regulation and introjection (controlled motivations), and identified and intrinsic regulations (autonomous motivations), with three items per motivation regulation (e.g., intrinsic motivation; “I exercise because it’s fun”). To improve readability, the 5-point Likert scale was amended from (0—not true for me, 1—not really true for me, 2—sometimes true for me, 3—often true for me, 4—very true for me) to (0—really not true for me, 1—not true for me, 2—sometimes true for me, 3—true for me, 4—really true for me). 

A bifurcation approach as described by Wilson, Sabiston, Mack, and Blanchard [37] was utilised to calculate composite scores for autonomous motivation (Σ((Intrinsic motivation) + (Identified regulation))/2) and controlled motivation (Σ((Introjection) + (External regulation))/2). A reliability analysis revealed good internal consistency for autonomous motivation (*α* = 0.88), whereas controlled motivation showed lower internal consistency (*α* = 0.65). Investigation into the controlled motivation subscale revealed good internal consistency for external regulation (*α* = 0.74) but unacceptable internal consistency for introjection (*α* < 0.50). The measurement of introjection in children has shown mixed results in terms of internal consistency. Some researchers have suggested that this may be due to difficulty in differentiating between external regulation and introjection in children [36]. As in other child studies [30], introjection was thus excluded from analysis and external regulation was used as a measure of controlled motivation.

#### 2.2.3. Emotional and Behavioural Difficulties

Parents completed the parent-report Strengths and Difficulties Questionnaire (SDQ) [38] to assess their child’s level of emotional and behavioural difficulties. The SDQ is a widely used mental health measure in children and adolescents aged 4–17 years [39]. It comprises 25 items, measuring psychological/behavioural attributes on a three-point Likert scale ranging from 0 (not true) to 2 (certainly true). The present study assessed the SDQ total score, which ranges from 0–40 with higher values indicating greater behavioural and emotional problems.

### 2.3. Data Analysis

Data were examined for outliers and any violations of multivariate analyses assumptions [40]. Inspection of residual partial plots indicated the presence of three outliers which were removed from the sample. All other assumptions of multivariate analyses were satisfied. The final sample comprised 87 participants. Descriptive statistics and bivariate Pearson correlations of the study variables were computed, excluding gender, for which independent *t*-tests were computed. To ascertain whether autonomous motivation for exercise explained variance in mental health (as measured by SDQ total score) and variance in PA levels (as measured by MVPA minutes), two separate standard regression models were conducted. The first model considered the variance in SDQ scores, attending to this study’s primary aim. The second model considered variance in MVPA, in line with the secondary aim of this study. Evidence shows age and gender are associated with children’s PA levels [18,41,42] and age with SDQ scores [18], thus, these variables were included in both models as covariates. MVPA was added as a covariate in the regression considering SDQ scores. 

In light of the nested sampling design, data were inspected visually to check for potential clustering effects at school level (using boxplots and scatterplots modelling our variables and associations of interest separately at each school). Analyses were further rerun with school as a covariate to assess the possible impact of the nested sampling design on regression models. Neither visual inspection of our data, nor the inclusion of school as a covariate, suggested meaningful clustering at school level; consequently, the hierarchical structure of our data was not explicitly modelled.

## 3. Results

Table 1 shows participant characteristics and descriptive statistics for the final sample (*n =* 87). The parent-reported data indicated that most children (56.32%) met the World Health Organization’s (WHO) PA guidelines of at least 60 min of MVPA per day (>419 min per week) [43]. Three percent of children’s SDQ scores were within the abnormal range, indicating these children were experiencing significant emotional and behavioral problems. An additional three percent of children’s SDQ scores were within the borderline range, indicating a greater likelihood of significant problems than those with lower scores. 

Table 2 presents bivariate Pearson correlations showing associations among the study variables. Independent-sample *t*-tests found that mean values between males and females did not differ for MVPA levels (*M[SD]_male_* = 483.60 [293.76]; *M[SD]_female_* = 440.23 [309.82]; *t = 0*.67, *df =* 85, *p* = 0.51) or SDQ scores (*M[SD]_male_* = 7.00 [4.99]; *M[SD]_female_* = 7.16 [4.13]; *t* = −0.16, *df* = 85, *p* = 0.87). The first regression analysis assessed the association between autonomous motivation and external regulation with SDQ scores, adjusting for MVPA, age and gender. The overall model explained a total of 8% variance in SDQ scores, (*R*^2^ = 0.14, *F* [5,84] = 2.49, *p* = 0.038, *adjR*^2^ = 0.08) (see Table 3). The individual effect of autonomous motivation was significant, showing a negative relationship with SDQ scores (*β* = −0.25, *p* = 0.030), whereas the individual effects of age, gender, MVPA and external regulation were nonsignificant. 

A second regression analysis was conducted to assess the association between autonomous motivation and external regulation with MVPA levels, adjusting for age and gender (see Table 4). The overall model was statistically significant and explained a total of 10% variance in MVPA levels, (*R*^2^ = 0.14, *F* [4,84] = 3.34, *p* = 0.014, *adjR*^2^ = 0.10). The individual effects of age, gender and external regulation were nonsignificant, whereas autonomous motivation shared a significant positive relationship with MVPA (*β* = 0.24, *p* = 0.034).

## 4. Discussion

The primary aim of this study was to examine the relationship between motivation for PA and children’s emotional and behavioural difficulties. The results supported the hypothesis that autonomous motivation is associated with fewer emotional and behavioural difficulties in a sample of Australian children. The secondary aim of this study was to examine the relationship between motivation for PA and children’s PA levels. Once again, the results supported the hypothesis that autonomous motivation is associated with higher levels of MVPA in a sample of Australian children.

In response to our primary aim, this study provides support for the self-determination theory notion that autonomous motivation will underpin improved mental health outcomes. However, not all studies have found significant results between autonomous motivation and mental health in children [22]. A nonrandomised controlled trial in a sample of children (*M*_age_ = 8.7 years) found no direct relationship between motivation and mental health [22]. The same study did, however, report a direct relationship between mental health and the satisfaction of basic needs (autonomy, competence, relatedness), the precursors of autonomous motivation. As such, the authors suggested the relationship between PA participation and wellbeing is nuanced and likely mediated by both motivation and needs-satisfaction.

External regulation was not associated with emotional and behavioural difficulties in the current study. When considered with the significant results of autonomous motivation, this confirms the hypothesis that autonomous motivation would be associated with fewer emotional and behavioural difficulties, whereas controlled motivation would not. This finding is congruent with the results reported by other studies (e.g., [29]). However, not all studies have reported a null effect for controlled motivation with mental health. Controlled motivation has been correlated with mental health problems in adults and adolescents, including increased symptoms of depression [28], increased negative affect [27] and decreased self-worth [44]. Whilst the present study’s null effect for external regulation was expected, the age of the current sample may partly explain the null result in comparison to studies in adolescents and adults that report a significant effect. Considering the social, biological and psychological developments that take place from childhood to adulthood [45], it is possible that the relationship between motivation for PA and mental health is influenced by age [46], whereby the positive relationship between controlled motivation and mental health problems strengthens with age. For example, children may be less likely to experience negative emotions when participating in PA that relate to external regulation factors (e.g., pleasing a parent or teacher), compared to an adolescent or adult. These results regarding the relationship between motivation for PA and children’s mental health are encouraging, as they suggest that interventions that focus on fostering children’s genuine interest in PA over competition or rewards may support improved mental health outcomes.

Studies investigating motivation for PA in children remain underrepresented. Thus, the secondary aim of this study was to explore the relationship between motivation for PA and levels of MVPA within the Australian context. A positive relationship between autonomous motivation and MVPA was found in the regression model, whereas the model did not support a relationship between external regulation and MVPA. These results indicate that children who either enjoy or value PA are more likely to be active than children who are motivated by rewards or competition. Interestingly, external regulation was found to negatively relate to MVPA levels in the bivariate correlation analysis. This finding may indicate the presence of a confounding variable (e.g., children’s competence levels). Lower perceptions of competence may contribute to lower PA levels and higher external regulation in children [47], thus explaining the bivariate association between external regulation and PA levels. Alternatively, research of a school-based PA program suggested that maladaptive relationships between controlled motivation and PA levels can be moderated by high levels of autonomous motivation [48]. For example, a child with high levels of both controlled and autonomous motivation may still choose to participate in PA because they enjoy it, but also be highly motivated by external rewards. Therefore, the balance between the motivation types, or basic needs, may be equally important in understanding the relationship between motivation for PA and children’s participation in PA. Whilst further research is needed to understand these interrelationships, future interventions seeking to increase children’s PA levels may wish to consider the role of autonomous motivation.

Given the multidimensional nature of both mental health [49] and PA participation [50], the relatively small magnitude of variance explained for both models was not surprising. A number of psychological factors have been shown to be associated with a child’s mental health status such as self-esteem [51], self-compassion [51] and resilience [52]. Further, these psychological factors should be considered within a broader biopsychosocial model [53]. Other well-established precursors include, but are not limited to, parental mental health [54], past adverse childhood experiences [55] and socio-economic status [56]. Likewise, participation in PA is influenced by a complex system of psychological, interpersonal and environmental factors [50]. Whilst many of these factors are difficult (e.g., socio-economic status) or unable (e.g., past adverse childhood experiences) to be modified, studies indicate a child’s motivation for PA can be shifted with intervention [57]. Thus, as a factor that can be modified, motivation for PA represents an important potential lever to be utilised in future programs aimed at improving children’s mental and physical health.

Although research into self-determined motivation and mental health is becoming more popular, studies predominantly utilise adolescent or adult samples [32]. The present study is one of the first to investigate the relationship between motivation for PA and mental health in children, offering preliminary evidence that motivation for PA, in particular autonomous motivation, plays a role in children’s mental health. Secondly, the current findings contribute to the research base investigating motivation for PA in Australian children aged 8 to 12 years, which has been lacking in the literature.

The findings should be interpreted in light of some limitations in the study. One limitation is related to the subjective measurement of MVPA. Subjective measurements of MVPA, while cost and time effective, are more vulnerable to error via biases such as social desirability and recall compared to objective measures such as accelerometers [58,59]. Additionally, whilst all children in participating grades were invited to participate in the study, class participation was at the discretion of the school principal and teachers. As such, the sample may overrepresent particular classes, schools and parents that value student wellbeing compared to the broader population.

The findings of this study support an association between autonomous motivation and mental health. However, due to the relatively small sample, further research is needed to replicate the findings in larger samples. Additionally, while the results suggest that autonomous motivation for PA is associated with reduced mental health problems, the size of the effect was relatively small. Further, the cross-sectional nature of this study precludes causal inferences. Thus, research testing motivation for PA alongside other well-established determinants of mental health in experimental and/or longitudinal designs is necessary.

## 5. Conclusions

The present study offers a preliminary investigation into the relationship between motivation for PA and mental health in children aged 8–12 years. The results indicate that children who are autonomously motivated to participate in PA may be less likely to experience emotional and behavioural difficulties. This finding is important, as it suggests that interventions that focus on fostering children’s genuine interest in PA over competition or rewards may improve both PA and mental health outcomes. Further research of child-specific interventions is needed to better understand the interactions between motivation, PA and mental health to ascertain whether motivation can be leveraged to improve children’s health outcomes in the future.

## Figures and Tables

**Table 1:** Participant characteristics and descriptive statistics.

Variable	*N or (M)*	% or (SD)
Age in years	(10.02)	(1.09)
School grade		
Grade 3	19	21.84
Grade 4	16	18.39
Grade 5	35	40.23
Grade 6	17	19.54
Gender		
Male	43	49.43
Female	44	50.57
Diagnosed mental disorder		
Anxiety or depression	1	1.15
AD/HD	2	2.30
ASD	3	3.45
MVPA minutes/week	(461.67)	(307.01)
Meeting WHO guideline (>419 min/week)	49	56.32
Not meeting WHO guideline (<419 min/week)	38	43.68
SDQ total score	(7.08)	(4.55)
Normal range (0–13)	81	93.10
Borderline range (14–16)	3	3.45
Abnormal range (17–40)	3	3.45

Note: AD/HD = Attention Deficit/Hyperactivity Disorder, ASD = Autism Spectrum Disorder, MVPA = Moderate to Vigorous Physical Activity, WHO = World Health Organization, SDQ = Strengths and Difficulties Questionnaire.

**Table 2:** Bivariate Pearson correlations among variables.

Variable	1	2	3	4	5
(1) Age	-				
(2) MVPA mins/week	0.16	-			
(3) Autonomous motivation	0.15	0.32 **	-		
(4) External regulation	0.16	−0.23 *	−0.29 **	-	
(5) SDQ total	−0.10	−0.22 *	−0.32 **	0.18	-

Note. * *p* < 0.05, ** *p* < 0.01.

**Table 3:** Simultaneous multiple regression analysis exploring the relationship between Strengths and Difficulties Questionnaire (SDQ) total score and Behavioural Regulation in Exercise Questionnaire (BREQ) autonomous motivation and external regulation for exercise scores.

Variable	B (95% CI)	SE B	*β*	*p*
Constant	16.75 ** (6.51, 26.99)	5.15		0.002
Age	−0.25 (−1.18, 0.69)	0.47	−0.06	0.601
Gender	−0.29 (−2.23, 1.66)	0.98	−0.03	0.771
MVPA	−0.01 (−0.02, 0.01)	0.01	−0.13	0.255
Autonomous motivation	−1.98 (−3.76, −0.19)	0.90	−0.25 *	0.030
External regulation	0.47 (−0.69, 1.62)	0.58	0.09	0.424

Note: *R*^2^ = 0.14, *p* = 0.038, *adjR*^2^ = 0.08. B = unstandardized coefficient, 95% CI = 95% confidence intervals, SE = standard error, **β** = standardized coefficient. * *p* < 0.05, ** *p* < 0.01.

**Table 4:** Simultaneous multiple regression analysis exploring the relationship between Moderate-to-vigorous Physical Activity (MVPA) and Behavioural Regulation in Exercise Questionnaire (BREQ) autonomous motivation and external regulation for exercise scores.

Variable	B (95% CI)	SE B	*β*	*p*
Constant	−301.12 (−970.81, 368.57)	336.51		0.374
Age	42.39 (−18.11, 102.89)	30.40	0.15	0.167
Gender	−3.42 (−131.44, 124.61)	64.33	−0.01	0.958
Autonomous motivation	123.98 (9.90, 238.07)	57.33	0.24 *	0.034
External regulation	−62.10 (−136.60, 12.41)	37.44	−0.18	0.101

Note. *R*^2^ = 0.14, *p* = 0.014, *adjR*^2^ = 0.10. B = unstandardized coefficient; 95% CI = 95% confidence intervals, SE = standard error, *β* = standardized coefficient. * *p* < 0.05.
